# Classifying individual differences in interoception: Implications for the measurement of interoceptive awareness

**DOI:** 10.3758/s13423-019-01632-7

**Published:** 2019-07-03

**Authors:** Jennifer Murphy, Caroline Catmur, Geoffrey Bird

**Affiliations:** 1 Social, Genetic and Developmental Psychiatry Centre, Institute of Psychiatry, Psychology and Neuroscience, King’s College London, PO80, De Crespigny Park, Denmark Hill, London, SE5 8AF UK; 2 Department of Psychology, Institute of Psychiatry, Psychology and Neuroscience, King’s College London, London, UK; 3 Department of Experimental Psychology, University of Oxford, Oxford, UK

**Keywords:** Interoceptive accuracy, Interoceptive attention, Interoceptive sensibility, Interoceptive awareness, Interoception

## Abstract

It has been suggested that individual differences in interoception (the perception of the body’s internal state) can be divided into three distinct dimensions: interoceptive accuracy (performance on objective tests of interoceptive accuracy), interoceptive sensibility (self-reported beliefs concerning one’s own interoception) and interoceptive awareness (a metacognitive measure indexed by the correspondence between interoceptive accuracy and interoceptive sensibility). Research conducted under this model underscores the importance of interoceptive awareness for a variety of disorder-specific and transdiagnostic symptoms. However, the clinical importance of interoceptive awareness means that this aspect of interoception warrants further scrutiny, and such scrutiny suggests that revision of the three-dimensional model of interoception is necessary. In this theoretical paper, we outline such a revision, highlighting a need to distinguish not only how interoception is measured (objective measures vs. self-report), but also what is measured (accuracy vs. attention). The model refines how individual differences in interoception are categorised, with important consequences for the measurement of interoceptive awareness. Such a revision may help researchers to identify the strengths and weaknesses in interoception observed across clinical conditions, and to isolate clinically relevant individual differences.

## Introduction

Theories linking individual differences in interoception (perception of the body’s internal state; Craig, 2003; Khalsa et al., 2018) to individual differences in cognitive ability and affective function, and to physical and mental health, are becoming increasingly common (e.g. Brewer, Happé, Cook, & Bird, 2015; Garfinkel, Seth, Barrett, Suzuki, & Critchley, 2015; Khalsa et al., 2018; Murphy, Catmur, & Bird, 2018c; Quattrocki & Friston, 2014). Given the increased focus on interoception, however, there is a growing need for a classification framework that categorises the various ways individuals may differ with respect to interoception. Perhaps the most well-known model (Garfinkel et al., 2015) proposes that interoception is a three-dimensional construct, comprising (1) interoceptive accuracy (as measured by performance on objective measures of interoception; e.g. heartbeat counting or detection tasks; Dale & Anderson, 1978; Katkin, Reed, & Deroo, 1983; Schandry, 1981; Whitehead, Drescher, Heiman, & Blackwell, 1977); (2) interoceptive sensibility (self-reported beliefs concerning one’s own interoception; measured using confidence ratings or questionnaires); and (3) interoceptive awareness (a metacognitive measure reflecting the correspondence between interoceptive accuracy and interoceptive sensibility, also referred to as interoceptive insight; Khalsa et al., 2018). Adoption of this model by a number of empirical studies has resulted in increased recognition of the importance of interoceptive awareness, with the correspondence between interoceptive accuracy and interoceptive sensibility emerging as a clinically relevant feature across a number of different disorders (Garfinkel et al., 2016; Paulus & Stein, 2006, 2010; Rae, Larsson, Garfinkel, & Critchley, 2018), and being predictive of certain transdiagnostic psychiatric symptoms (Ewing et al., 2017). The clinical importance of interoceptive awareness means that this aspect of interoception deserves further scrutiny, and such scrutiny may require the three-dimensional model of interoception to be revised. These issues are the focus of this theoretical paper.

Interoceptive awareness is quantified by examining the correspondence between measures of interoceptive accuracy and interoceptive sensibility. As such, the measurement of interoceptive awareness depends upon the degree to which both interoceptive accuracy and interoceptive sensibility can be measured accurately, and how these measures are combined. Although the validity of certain measures of interoceptive accuracy are debated (e.g. Desmedt, Luminet, & Corneille, 2018; Khalsa, Rudrauf, Sandesara, Olshansky, & Tranel, 2009; Murphy, Brewer, Hobson, Catmur, & Bird, 2018a; Zamariola, Maurage, Luminet, & Corneille, 2018), the desirable qualities of a good measure are fairly self-evident: The test should measure the accuracy of perception of an unambiguously interoceptive signal by reference to an objective measure of that signal. In contrast, interoceptive sensibility is concerned with one’s self-reported beliefs regarding one’s “dispositional tendency to be internally self-focused and interoceptively cognisant” (Garfinkel et al., 2015) and is typically measured using questionnaire measures such as the Porges Body Perception Questionnaire (BPQ; Porges, 1993) or confidence ratings during a task of interoceptive accuracy (Ehlers, Breuer, Dohn, & Fiegenbaum, 1995). Problematically, scores on these two commonly used measures of interoceptive sensibility (i.e. questionnaire measures and confidence ratings) are not usually correlated with each other (e.g. Garfinkel et al., 2015; Murphy, Brewer, Plans, et al., 2018b). Furthermore, they have been reported to show differential relationships with interoceptive accuracy: whilst confidence ratings sometimes correlate with interoceptive accuracy, questionnaire measures like the BPQ typically do not (e.g. Critchley, Wiens, Rotshtein, Ohman, & Dolan, 2004; Ferentzi, Drew, Tihanyi, & Köteles, 2018; Garfinkel et al., 2015; Murphy, Brewer, Plans et al., 2018b; though this may depend on the measure of interoceptive accuracy employed; see Forkmann et al., 2016; Garfinkel et al., 2015; Schulz, Lass-Hennemann, Sütterlin, Schächinger, & Vögele, 2013). Nevertheless, as interoceptive sensibility and interoceptive accuracy are not always correlated, such findings have been taken as evidence that interoceptive accuracy and interoceptive sensibility are distinct and dissociable (e.g. Garfinkel et al., 2015).

Interoceptive awareness is typically calculated by assessing the correspondence between objectively measured interoceptive accuracy using a specific test, and confidence judgements relating to performance on that test (Garfinkel et al., 2015). More recently, however, researchers have begun to examine the correspondence between interoceptive accuracy and interoceptive sensibility by using the BPQ as a measure of interoceptive sensibility (Garfinkel et al., 2016; Rae et al., 2018). Although this is typically referred to as ‘trait interoceptive prediction error’ (TIPE) rather than interoceptive awareness, within the three-dimensional model of interoceptive ability TIPE must be a variant of interoceptive awareness because it indexes the correspondence between interoceptive accuracy and interoceptive sensibility. However, whilst TIPE and interoceptive awareness ostensibly measure the same thing (the correspondence between interoceptive accuracy and interoceptive sensibility) and have both been highlighted as clinically relevant (Ewing et al., 2017; Garfinkel et al., 2016; Rae et al., 2018), they appear to be distinct; for example, whilst TIPE has been found to be atypical in people with autism spectrum disorder, interoceptive awareness has not (Garfinkel et al., 2016).

**Fig. 1:**
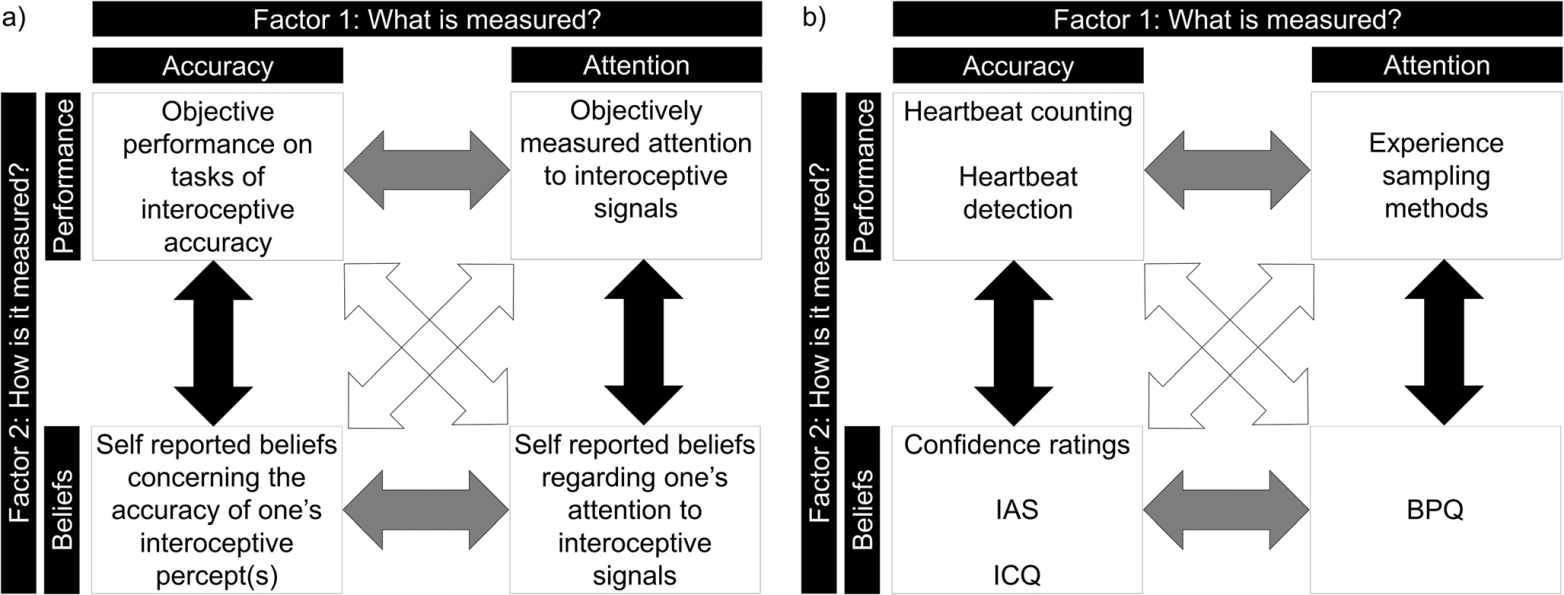
Model of interoceptive ability. (**a**) 2 × 2 factorial model of interoceptive abilities. Factor 1 distinguishes whether accuracy or attention is the target of measurement. Factor 2 distinguishes whether a measure of objective performance or a self-report measure of beliefs is utilised. Four facets are therefore defined: (1) objective interoceptive accuracy; (2) self-reported beliefs concerning one’s interoceptive accuracy; (3) objective interoceptive attention; and (4) self-reported beliefs concerning one’s interoceptive attention. For both accuracy and attention, interoceptive awareness can be quantified by comparing one’s self-reported beliefs to the objective measure (black arrows). Correspondence across measures within the same measurement factor can be quantified (grey arrows) as well as the relationship across different measurement and performance factors (white arrows). (**b**) Illustrative tasks that may index distinct facets of the model. *IAS* Interoceptive Accuracy Scale (Murphy et al., 2018; e.g. items such as “I can always accurately perceive when my heart is beating fast”). *ICQ* Interoceptive Confusion Questionnaire (e.g. items such as “I am very sensitive to changes in my heart-rate”; Brewer et al., 2016). *BPQ* Porges Body Perception Questionnaire (items such as “during most situations I am aware of how hard my heart is beating”; Porges, 1993)

Given this ambiguous relationship between interoceptive awareness and TIPE, and their clinical relevance, there is a clear need for a theoretical model that can distinguish between the various ways individuals may differ with respect to interoception. Such a model would allow for greater precision when categorising the patterns of strengths and weaknesses in interoception across different psychiatric conditions, and may also facilitate more specific interventions. We therefore suggest a modification of the existing three-dimensional model of interoception, arguing instead for a 2 × 2 factorial model. The first factor refers to which of the two main features of interoceptive perception is the target of measurement: accuracy versus attention^1^ (Fig. 1). Here, accuracy refers to the degree to which one’s interoceptive perception is a veridical representation of the true state of the body, while attention refers to the degree to which interoceptive signals are the object of attention. The second factor relates to the type of measurement: objective versus self-report (of course, such a distinction also affects that which is measured; e.g. an individual’s interoceptive accuracy vs. their perception of their interoceptive accuracy). Such a 2 × 2 model gives rise to four core measures of interoceptive ability: (1) objective measurement of the accuracy of interoceptive perception (e.g. performance on objective measures of interoception such as the heartbeat tracking or detection procedures); (2) self-reported perception of interoceptive accuracy (i.e., one’s beliefs regarding the accuracy of one’s interoceptive percept, including confidence ratings (e.g. ratings on a visual analogue scale from ‘full perception/complete confidence’ to ‘total guess/no awareness’) or scores on questionnaires such as the Interoceptive Confusion Questionnaire or Interoceptive Accuracy Scale, for example items such as “I can always accurately perceive when my heart is beating fast”; Brewer, Cook, & Bird, 2016; Murphy, Brewer, Plans et al., 2018b); (3) objective interoceptive attention (e.g. objective measurement of the degree to which interoceptive signals are the object of attention, such as experience sampling methods^2^; see Csikszentmihalyi & Larson, 2014) and (4) self-reported interoceptive attention (one’s beliefs regarding the degree to which interoceptive signals are the object of attention, for example the BPQ; e.g. items such as “during most situations I am aware of how hard my heart is beating”; Porges, 1993).

Like the three-dimensional model, therefore, our model highlights the importance of distinguishing between *how* interoception is measured (e.g. objectively or via self-report) but also incorporates *what* is being measured (e.g. attention or accuracy) in order to distinguish possible individual differences in interoception. Crucially, such a distinction holds important consequences for measurement of interoceptive awareness. In the existing three-dimensional model interoceptive awareness refers to the correspondence between measures of interoceptive accuracy and interoceptive sensibility – regardless of whether the measure of interoceptive sensibility relates to one’s perception of the accuracy of interoceptive perception (e.g. confidence ratings) or one’s beliefs regarding one’s degree of attention to interoceptive signals (e.g. the BPQ). However, as noted, existing data suggest that these two ‘interoceptive awareness’ measures appear to quantify distinct aspects of interoception; for example, they often show differential associations with symptomology and differential patterning across disorders (Garfinkel et al., 2016; Rae et al., 2018). In accordance with this distinction, the 2 × 2 factorial model described above makes clear that it is possible to calculate two distinct metacognitive (correspondence) measures (Fig. 1, black arrows): (1) the correspondence between objectively and subjectively measured interoceptive accuracy (‘awareness of interoceptive accuracy’),^3^ and (2) the correspondence between objectively and subjectively measured interoceptive attention (‘awareness of interoceptive attention’). Note that this suggestion does not invalidate existing studies which utilise the correspondence between subjective measurement of interoceptive attention (e.g. the BPQ) and objective measurement of interoceptive accuracy (e.g. tasks of cardiac interoceptive accuracy), or negate the demonstrated clinical utility of such a measure (which is indicated by the white arrows in Fig. 1). Rather, it provides a conceptual framework within which the different measures of interoceptive awareness may be distinguished, and highlights that an accuracy : attention correspondence measure does not meet the typical requirement for a metacognitive measure – that the correspondence is calculated between objective and subjective measures of the same thing (e.g. objective measurement of interoceptive accuracy and subjective perception of interoceptive accuracy; e.g. Fleming & Dolan, 2012).

Such a framework for quantifying individual differences in interoception goes some way to explain the mixed results in the literature concerning the relationship between different measures of interoceptive sensibility, and the relationship between interoceptive accuracy and interoceptive sensibility, as reported inconsistences align with distinctions proposed by the 2 × 2 factorial model. For example, self-report measures of interoceptive attention (e.g. the BPQ) are not usually correlated with self-report measures of interoceptive accuracy (e.g. confidence ratings or questionnaires of interoceptive accuracy; Garfinkel et al., 2015; Murphy, Brewer, Plans et al., 2018b), although different self-report measures of interoceptive accuracy usually show some correspondence with each other (Murphy, Brewer, Plans et al., 2018b). One’s beliefs regarding interoceptive attention and one’s beliefs regarding interoceptive accuracy therefore appear distinct, an observation not captured by the current model that combines these measures under the heading of interoceptive sensibility. Likewise, it has been argued that there is typically little relationship between objective interoceptive accuracy and interoceptive sensibility (Garfinkel et al., 2015). However, existing data suggest that the relationship between objectively measured interoceptive accuracy and self-report measures of interoception may differ depending on whether the self-report measure assesses accuracy or attention; objectively measured interoceptive accuracy is sometimes associated with one’s self-reported beliefs regarding interoceptive accuracy, but not with one’s self-reported attention to interoceptive signals (e.g. Garfinkel et al., 2015; Murphy, Brewer, Plans et al., 2018b).

As well as providing a potential explanation for mixed results in the literature, such a revision may help researchers to identify the strengths and weaknesses in interoception observed across clinical conditions, and to isolate clinically-relevant individual differences. For example, an individual with atypical TIPE (heightened attention relative to accuracy) may benefit from different treatment than an individual with atypical awareness of interoceptive accuracy (confidence-accuracy relationship). Whilst the exact patterning of interoceptive processing across disorders remains a question for future research, this framework may help to conceptualise potential differences across disorders and, in the future, may be useful for translating these findings to clinical practice.

It is important to acknowledge, of course, that other aspects of interoception that are not captured by existing models may hold clinical relevance. For example, individuals may differ with respect to the extent that they use interoceptive signals in their everyday lives, in addition to the extent to which they can accurately perceive interoceptive signals and the extent to which interoceptive signals are the object of attention. Likewise, individuals may also differ in terms of how unified their interoceptive attention and/or accuracy is across different interoceptive signals (for example, an individual may be extremely good at perceiving cardiac signals, but poor at perceiving respiratory or gastric sensations). Moreover, it is indeed possible that the relationships between the facets of interoception outlined in our 2 × 2 model may differ depending on the interoceptive signal of interest (e.g. cardiac vs. gastric). At present, our understanding of the clinical relevance of these additional aspects of interoception, and the relationship between facets of interoception across interoceptive signals, is limited by the paucity of tests designed to assess these possible individual differences. However, further work may highlight a need to include additional aspects of interoception within this 2 × 2 model.

In summary, with growing interest in interoception, there is a need for a framework that adequately distinguishes between the various individual differences in interoception. The 2 × 2 factorial model provides a refinement of the existing model of interoceptive abilities, separating both whether interoceptive accuracy or attention is the target of measurement and how interoception is measured. It highlights the existence of two distinct interoception-related metacognitive measures, and explains mixed results in the literature. It is hoped that use of this model will allow researchers to identify the strengths and weaknesses in interoception observed across clinical conditions, and to isolate the clinically-relevant individual differences in interoception.

## Footnotes

1 Note that in previous descriptions of the model we used the term *awareness* rather than *attention* to refer to the degree to which interoceptive signals are the object of attention. However, given that the word *awareness* has been used to refer to a number of different aspects of interoception in the past, to avoid unnecessary confusion we use the term *attention* here.

2 Note that an objective measure of interoceptive attention may rely on self-report (e.g., in an experience-sampling procedure the participant might be repeatedly asked what is the object of their attention over the course of a day, week or month), but the proportion of time that interoceptive signals are the object of attention (relative to the proportion of time exteroceptive signals were the object of attention) can then be objectively determined.

3 One’s perception of, for example, one’s interoceptive accuracy may be measured using confidence ratings specific to a particular interoceptive signal at a particular point in time, or by trait measures assessing perceived accuracy across multiple interoceptive signals and across time. Accordingly, although awareness of interoceptive accuracy can be calculated using both measures, calculations using a trait measure would be less specific (with respect to interoceptive signal and time-point) than those using confidence judgements.
